# The Relationship between Subnormal Peak-Stimulated Growth Hormone Levels and Auxological Characteristics in Obese Children

**DOI:** 10.3389/fendo.2014.00035

**Published:** 2014-03-25

**Authors:** Jefferson Barrett, Louise Maranda, Benjamin Udoka Nwosu

**Affiliations:** ^1^Department of Pediatrics, University of Massachusetts Medical School, Worcester, MA, USA; ^2^Department of Quantitative Health Sciences, University of Massachusetts Medical School, Worcester, MA, USA

**Keywords:** growth hormone deficiency, obese children, growth hormone stimulation test, short stature, growth velocity, bone age, mid-parental target height

## Abstract

**Context:** The hypothesis that obese children are overdiagnosed with growth hormone deficiency (GHD) has not been adequately investigated in the context of adiposity-related differences in auxology.

**Aim:** To investigate the differences in auxological parameters between short, prepubertal, obese children, and normal-weight peers who underwent growth hormone stimulation testing (GHST).

**Hypothesis:** Over-weight/obese children with GHD [peak growth hormone (GH) < 10 μg/L] will have higher values for growth velocity (GV) standard deviation score (SDS), bone age minus chronological age (BA − CA), and child height SDS minus mid-parental height (MPTH) SDS when compared to normal-weight GHD peers.

**Subjects and Methods**: A retrospective review of anthropometric and provocative GHST data of 67 prepubertal, GH-naïve children of age 10.21 ± 2.56 years (male *n* = 45, age 10.8 ± 2.60 years; female *n* = 22, age 8.94 ± 2.10). Inclusion criteria: GHST using arginine and clonidine. Exclusion criteria: hypopituitarism, abnormal pituitary magnetic resonance imaging scan, syndromic obesity, or syndromic short stature. Data were expressed as mean ± SD.

**Results:** The over-weight/obese children with peak GH of <10 μg/L had significantly lower value for natural log (ln) peak GH (1.45 ± 0.09 vs. 1.83 ± 0.35, *p* = 0.022), but similar values for GV SDS, insulin-like growth factor-I, insulin-like growth factor binding protein-3, bone age, BA − CA, MPTH, and child height SDS minus MPTH SDS compared to normal-weight peers with GHD. After adjusting for covariates, the over-weight/obese children (BMI ≥ 85th percentile) were >7 times more likely than normal-weight subjects (BMI < 85th percentile) to have a peak GH of <10 μg/L, and 23 times more likely to have a peak GH of <7 μg/L (OR = 23.3, *p* = 0.021). There was a significant inverse relationships between BMI SDS and the ln of peak GH (β = −0.40, *r*^2^ = 0.26, *p* = 0.001), but not for BMI SDS vs. GV SDS, ln peak GH vs. BA, or ln peak GH vs. GV SDS.

**Conclusion:** Subnormal peak GH levels in obese prepubertal children are not associated with unique pre-GHST auxological characteristics.

## Introduction

Obese children are generally taller than their non-obese peers as demonstrated in studies carried out in the United States (1), Australia (2), and Japan (3). These studies show that during prepubertal years, obese children present with higher growth velocity (GV) standard deviation score (SDS) and accelerated bone age (BA) compared to non-obese children (4). However, studies that evaluated the relationship between obesity and the diagnostic tests for growth hormone (GH) deficiency (GHD), the GH stimulation tests (GHST), had suggested that obese children have subnormal peak GH response to GH secretagogues (5, 6). This has led to the hypothesis that obese children are overdiagnosed with GHD (5–7), which suggests a high prevalence of false-positive results in obese children diagnosed with GHD.

However, the effect of adiposity on GV and other auxological parameters has not been fully studied in children undergoing GHST. Such a study may provide additional data to reduce the rate of false-positive results from GHST, and improve the accuracy of the diagnosis of GHD given that the current diagnostic gold standard for GH deficiency, the GHST, has several drawbacks (8). These non-physiological tests have poor reproducibility and rely on GH assays of variable accuracy. Their results are influenced by circulating levels of sex steroids such that some researchers have recommended prior sex-steroid priming of prepubertal subjects undergoing GHST to prevent false-positive results (9). These tests may not discriminate between normal short children and children with partial GHD (8). GHSTs have a high false-positive rate, such that up to 25–67% of children diagnosed as GHD will have a normal peak GH when retested upon the completion of their GH therapy (10–12). As a result of the above limitations, a careful analysis of height, GV, insulin-like growth factor-I (IGF-I), and insulin-like growth factor binding protein-3 (IGFBP-3) and a search for an etiology are crucial for an accurate diagnosis (13). The current universally accepted cut-off value for the diagnosis of GHD by GHST is a peak-stimulated GH of <10 μg/L (14, 15).

Despite the limitations of the GHST, and the fact that the studies that reported diminished peak GH response in over-weight and obese children undergoing GHST (5, 6) suggested that the diminution in response could lead to an overdiagnosis of GHD in these subjects (7), it is unclear whether there are auxological, laboratory, and radiological features that could identify the over-weight/obese children who would otherwise have a false-positive result for GHD. For example, there is a dearth of data on the effect of obesity on GV in children undergoing GHST.

Given the above-mentioned limitations of GHST, and the fact that adiposity accounts for about 20% of the variability in peak GH response (5, 7), we designed this study to investigate the relationships between the reduced GH response to secretagogues in over-weight/obese children and their auxological, biochemical, and radiological parameters when compared to normal-weight individuals.

We hypothesized that over-weight/obese children with GHD will have higher values for GV SDS, BA minus chronological age (BA − CA), and child height SDS minus mid-parental target height (MPTH) SDS when compared to their normal-weight GHD peers. The study’s primary aim was to determine whether these short obese children with peak GH of <10 μg/L differed from short normal-weight children with peak GH of <10 μg/L on one hand, and over-weight/obese children with peak GH of >10 μg/L on the other. We further examined the above relationships using a cut-off peak GH response of <7 and <5 μg/L, respectively. The secondary aim was to determine the relationship between peak GH response and GV SDS.

## Subjects and Methods

### Subjects

The study was approved by the Institutional Review Board of the University of Massachusetts Medical School. The clinical records of prepubertal children (*n* = 67; 45 males and 22 females) of mean age 10.21 ± 2.56 years, were included if they had height below the fifth percentile and had undergone two unprimed GHST using clonidine and arginine, from 2004 to 2012. Five subjects were excluded because they either had a one-agent GHST, or had received other GH secretagogues such as GH releasing hormone (GHRH). Subjects were further excluded if they had hypopituitarism as diagnosed by biochemical studies showing subnormal pituitary hormone levels; abnormal results of magnetic resonance imaging (MRI) of the pituitary gland, history of medulloblastoma, pituitary adenoma or any systemic illness or syndrome affecting growth such as renal failure, or Turner syndrome, respectively. Three subjects with peak GH of <10 μg/L were excluded based on MRI criteria.

### Anthropometry

Standing height was measured with a Harpenden stadiometer (Holtain Ltd., Crymych, Dyfed, UK). Weight was determined using a Detecto scale (Detecto Scale Co., Webb City, MO, USA). Height, weight, and body mass index (BMI, weight/height^2^) were expressed as SDS for age and gender based on National Center for Health Statistics (NCHS) data (16). Gender-adjusted MPTH *z*-score was calculated for 18-year-old adults using NCHS data and the standard formula for MPTH (17). The MPTH is a child’s projected adult height based on the heights of his or her parents and is calculated as follows: for girls, the father’s height minus 13 cm (5″) is averaged with the mother’s height; for boys, the mother’s height plus 13 cm is averaged with the father’s height (17). Anthropometric data were expressed as mean ± SD. Heights of parents were obtained by history (18) and 16 (23.8%) by measurement in the clinic.

All GV data were obtained from at least two height measurements of at least 12-month interval, while making certain that all measurements occurred prior to the institution of GH therapy in subjects who went on to receive GH treatment. GV data were expressed as SDS based on established norms (19–21).

Pubertal status was established by pediatric endocrinologists using the method of Tanner and Marshall (22, 23). Prepubertal status was marked by a testicular volume of ≤3 cc in boys as measured with a Prader orchidometer, and Tanner stage 1 breasts in girls as indicated by the absence of breast buds or breast tissue. BA data in all patients were obtained within 6 months of GHST and read by the same pediatric radiologist.

### Biochemical and imaging studies

All subjects underwent two separate GHST, without sex-steroid priming, in a single day using clonidine (0.125 mg/m^2^ p.o.), and arginine (0.5 g/kg i.v.). Samples were obtained for GH determination at times 0, 15, 30, 60, 90, and 120 min according to established procedure (24).

### Assays

Serum IGFBP-3 levels were measured by radioimmunoassay (RIA) (Esoterix, Calabasas Hills, CA, USA). Its inter-assay coefficients of variation (CV) were 5.5, 7.8, and 18% (at 2.9, 2.7, and 1.0 mg/L), respectively. Intra-assay CV were 5.1 and 13% (at 2.7, and 1.0 mg/L), respectively. Its sensitivity was 0.3 mg/L.

Both serum GH and IGF-I levels were measured using a solid-phase, two-site chemiluminescent immunometric assay, Immulite 2000 (Siemens Healthcare Diagnostics, Deerfield, IL, USA). The GH assay was referenced to the World Health Organization Second International Standard 98/574 for somatotropin (22-kDa recombinant DNA-derived materials) at a standardized specific activity of 3.0 IU/mg (25). The GH assay had an analytical sensitivity of 0.01 μg/L, intra-assay CV of 2.9–4.6%, and inter-assay CV of 4.2–6.6%; while the IGF-I assay had an analytical sensitivity of 20 μg/L, intra-assay CV of 2.3–3.9%, and inter-assay CV of 3.7–8.1%.

Bone age was assessed using the Greulich and Pyle method (26). BA–CA was obtained by subtracting patient’s chronological age from his/her skeletal age.

### Statistical analysis

Statistical analyses were performed using the SPSS Predictive Analytics SoftWare v. 21 (IBM Corporation, Armonk, NY, USA). Means and SD were calculated for descriptive summary statistics and for GH, IGF-I, IGFBP-3, BA, BA–CA, and GV. Anthropometric and laboratory data were compared using Student’s *t*-test. The difference in the number of male children was calculated using Fisher’s exact test. Data were expressed as mean ± SD.

Multiple linear regression models were constructed to explore the adjusted relationship between BMI and peak GH, IGF-I, IGFBP-3, and GV SDS. Peak GH, IGF-I, and IGFBP-3 values were log-transformed to achieve normality. A multivariate logistic model was fitted to the data to explore the possible relationships between BMI and peak GH, adjusting for possible confounders.

For the analyses, subjects were first stratified by peak GH of <10 vs. ≥10 μg/L; <7 vs. ≥7 μg/L, and then by peak GH of <5 vs. ≥5 μg/L, to determine the differences in auxological parameters between the GHD and growth hormone sufficient (GHS) subjects using each of the above cut-off values. We evaluated the three cut-off values because of the arbitrary nature of the choice of the cut-off points for the diagnosis of GHD using GHST over the years. We further analyzed the auxological, biochemical, and radiological differences between the normal-weight and over-weight/obese subjects stratified by peak GH levels.

In a subsequent analysis, we stratified the subjects by BMI criteria to determine the differences in GV SDS and other auxological parameters between normal-weight (BMI < 85th percentile) and over-weight/obese subjects (BMI ≥ 85th percentile).

## Results

Table 1 shows the patients’ characteristics stratified by BMI status. Though the natural log (ln) of peak GH response was significantly lower in the over-weight/obese children compared to the normal-weight subjects (2.18 ± 0.82 vs. 2.83 ± 0.55 μg/L, *p* = 0.011), there were no significant differences between these groups for GV SDS (−1.39 ± 1.46 vs. −1.80 ± 2.7, *p* = 0.60), BA (*p* = 0.31), BA–CA (*p* = 0.97), MPTH (*p* = 0.95), and child height SDS minus MPTH SDS (*p* = 0.77).

**Table 1:** **Characteristics of prepubertal patients stratified by body mass index percentile**.

	Body mass index (kg/m^2^)		
Parameter	Normal weight (BMI < 85th percentile)	Over-weight/obese (BMI ≥ 85th percentile)	*p* Value
	*n* = 61	*n* = 6	
Age (years)	10.12 ±2.52	11.17 ± 3.05	0.446
Height SDS	−2.31 ±0.67	− 2.37 ± 0.33	0.734
Weight SDS	−1.84 ±0.85	− 0.18 ± 0.49	*****<***0.001**
BMI SDS	−0.54 ±0.85	1.27 ± 0.28	*****<***0.001**
Number of males (%)	39/61 (63.9%)	6/6 (100%)	0.167
IGF-1 (μg/L)	134.37 ±66.5	131.50 ± 40.25	0.877
IGF-I SDS	0.70 ±0.64	0.82 ± 0.88	0.753
ln IGF-1 (μg/L)	4.77 ±0.53	4.84 ± 0.29	0.620
IGFBP-3 (mg/L)	3.88 ±1.05	4.12 ± 0.74	0.372
ln IGFBP-3 (mg/L)	1.32 ±0.27	1.40 ± 0.19	0.145
Growth velocity SDS	−1.8 ± 2.7	−1.39 ±1.46	0.600
Bone age (BA) (years)	8.71 ±2.70	9.75 ± 2.14	0.310
BA–CA (years)	−1.40 ±1.44	− 1.38 ± 1.62	0.970
Peak GH response (μg/L)	19.54 ±12.37	11.50 ± 8.49	0.071
ln peak GH response (μg/L)	2.83 ±0.55	2.18 ± 0.82	**0.011**
MPTH SDS	−0.51 ±0.92	− 0.54 ± 0.87	0.954
Child height SDS – MPTH SDS	−1.83 ±1.11	− 1.70 ± 0.90	0.774

*SDS, standard deviation score; IGF-I, insulin-like growth factor-I; IGFBP-3, insulin-like growth factor binding protein-3; BA − CA, bone age minus chronological age; MPTH, mid-parental target height. Significant p values are bolded*.

When the subjects were first stratified by a peak GH level of 10 μg/L (Table 2), children with peak GH level of <10 μg/L (i.e., GHD) were older (11.8 ± 2.61 vs. 9.90 ± 2.45 years, *p* = 0.042), had higher BMI SDS (0.24 ± 1.03 vs. −0.50 ± 0.92, *p* = 0.044), and significantly lower value for IGF-I SDS (−1.18 ± 0.74 vs. −0.62 ± 0.60, *p* = 0.036) compared to the non-GHD children. There were no significant differences in the values for GV SDS, BA − CA, MPTH SDS, child height SDS minus MPTH SDS, or IGFBP-3 between the GHD and non-GHD groups.

**Table 2:** **Characteristics of prepubertal subject stratified by peak growth hormone level of 10 μg/L**.

Parameter	Peak GH ≥ 10	Peak GH < 10	*p* Value
	*n* = 56	*n* = 11	
Age (years)	9.90 ±2.45	11.81 ± 2.61	**0.042**
Height SDS	−2.32 ±0.67	− 2.30 ± 0.51	0.918
Weight SDS	−1.81 ±0.89	− 1.08 ± 1.07	0.054
BMI SDS	−0.50 ±0.92	0.24 ± 1.03	**0.044**
Number of males (%)	37/56 (66.1%)	8/11 (72.7%)	0.395
IGF-1 (μg/L)	137.79 ±68.09	114.10 ± 32.15	0.094
IGF-I SDS	−0.60 ±0.60	− 1.18 ± 0.74	**0.036**
ln IGF-1 (μg/L)	4.79 ±0.54	4.70 ± 0.32	0.433
IGFBP-3 (mg/L)	3.92 ±1.06	3.78 ± 0.82	0.639
ln IGFBP-3 (mg/L)	1.33 ±0.27	1.31 ± 0.23	0.780
Growth velocity SDS	−1.87 ±2.17	− 1.23 ± 4.31	0.675
Bone age (BA) (years)	8.53 ±2.58	10.14 ± 2.70	0.093
BA − CA (years)	−1.35 ±1.47	− 1.64 ± 1.37	0.542
MPTH SDS	−0.53 ±0.87	− 0.43 ± 1.14	0.820
Child height SDS – MPTH SDS	−1.79 ±1.04	− 2.00 ± 1.41	0.692

*SDS, standard deviation score; IGF-I, insulin-like growth factor-I; IGFBP-3, insulin-like growth factor binding protein-3; BA − CA, bone age minus chronological age; MPTH, mid-parental target height. Significant p values are bolded*.

Similarly, when the subjects were stratified by a peak GH level of 7 μg/L, children with peak GH level of <7 μg/L (i.e., GHD) were older (12.61 ± 2.56 vs. 9.88 ± 2.40 years, *p* = 0.02), had non-significantly higher BMI SDS (0.40 ± 1.18 vs. −0.48 ± 0.90, *p* = 0.076) and lower IGF-I SDS (−1.26 ± 0.86 vs. −0.62 ± 0.59, *p* = 0.099), but greater values for BA (11.21 ± 2.23 vs. 8.47 ± 2.54, *p* = 0.010), compared to the non-GHD children. There were no significant differences in the values for GV SDS (−0.56 ± 4.49 vs. −1.94 ± 2.22, *p* = 0.453), BA − CA (*p* = 0.953), MPTH SDS (*p* = 0.675), child height SDS minus MPTH SDS (*p* = 0.498), or IGFBP-3 (*p* = 0.809), between the GHD and non-GHD groups.

In a subsequent analysis, patients were stratified by a peak GH level of 5 μg/L. Subjects with peak GH of <5 μg/L were older (13.83 ± 2.45 vs. 9.92 ± 2.36, *p* = 0.021), had non-significantly lower values for IGF-I SDS (−1.50 ± 0.92 vs. −0.62 ± 0.59, *p* = 0.99), but greater values for BMI SDS (1.04 ± 0.60 vs. −0.49 ± 0.90, *p* = 0.002), and BA (12.4 ± 1.64 vs. 8.50 ± 2.50, *p* = 0.003), compared to those with peak GH level of ≥5 μg/L; but similar values for GV SDS (0.06 ± 6.15 vs. −1.91 ± 2.17, *p* = 0.569), BA − CA, MPTH SDS, child height SDS minus MPTH SDS, IGF-I SDS, and IGFBP-3.

After adjusting for age and gender, the over-weight/obese children were >7 times more likely than normal-weight subjects to have a peak GH of <10 μg/L [OR = 7.9 (95 CI 0.99–63.3) *p* = 0.051]; and >23 times more likely to have a peak GH of <7 μg/L [OR = 23.3 (95 CI 1.61–337.1), *p* = 0.021].

An analysis of the characteristics of the normal-weight and over-weight/obese subjects stratified by peak GH level of 10 μg/L showed that the over-weight/obese children with peak GH of <10 μg/L had significantly lower value for ln peak GH (1.45 ± 0.09 vs. 1.83 ± 0.35, *p* = 0.022), but similar values for GV SDS, IGF-I, IGFBP-3, BA, BA − CA, MPTH, and child height SDS minus MPTH SDS, as compared to normal-weight peers with GHD.

The analysis of the relationships between BMI SDS, ln peak GH response, and GV SDS showed that after adjusting for age and gender, there was a significant inverse relationship between BMI SDS and ln peak GH level (*r*^2^ = 0.26, β = −0.40, *p* = 0.001), but not between BMI SDS and GV SDS (*r*^2^ = 0.032, β = 0.11, *p* = 0.422). There were equally no relationship between ln peak GH and BA (*r*^2^ = 0.107, β = −0.11, *p* = 0.654), nor between ln peak GH and GV SDS (*r*^2^ = 0.164, β = −0.061, *p* = 0.643) after an initial adjustment for age and gender. However, when the above relationship between ln peak GH and GV SDS was further adjusted for BMI SDS, the *r*^2^ increased to 0.254 (β = −0.026, *p* = 0.835). A quadratic regression analysis showed a non-significant curvilinear relationship between BA and GV SDS (β_1_ = −0.956, β_2_ = 0.039; *r*^2^ = 0.0.061, *p* = 0.63) after adjusting for age, gender, and BMI SDS. Further analyses showed a non-significant positive association between BMI SDS and ln IGFBP-3 (*r*^2^ = 0.12, β = 0.166, *p* = 0.174); as well as BMI SDS and ln IGF-I (*r*^2^ = 0.287, β = 0.123, *p* = 0.257).

## Discussion

This study examined whether the hypothesis that short obese children were overdiagnosed with GHD had significant correlates with the GV and other auxological characteristics in these patients when compared to short normal-weight children undergoing GHST. Its central premise was that these obese children could demonstrate unique characteristics that differentiate them from short, normal-weight peers. These characteristics include significantly higher values for GV SDS, child height minus MPTH SDS, and BA − CA. In agreement with the above hypothesis, we found that over-weight/obese children had significantly lower peak GH response than normal-weight children. Furthermore, children with GHD had significantly greater BMI value than the non-GHD subjects at peak GH cut-off values of 10 and 5 μg/L, but not at 7 μg/L. There were, however, no significant differences in GV SDS between the obese and non-obese subjects, or between the subjects who were diagnosed with GHD or GH sufficiency. There were equally no differences in the values for MPTH SDS and child height SDS minus MPTH SDS between the GHD and non-GHD subjects; nor between the over-weight/obese and normal-weight subjects. Subjects with GHD had significantly lower values for IGF-I at a peak-stimulated GH cut-off value of 10 μg/L, and significantly higher values for BA at peak GH cut-off value of 5 and 7 μg/L, but not at 10 μg/L.

After adjusting for covariates, there was a significant inverse relationship between BMI SDS and ln peak GH level, but no relationships between BMI SDS and GV SDS; BMI SDS and ln IGF-I; BMI SDS and ln IGFBP-3; ln peak GH and BA; or GV SDS and BA.

Further analysis showed no differences in GV SDS, MPTH SDS, child height SDS minus MPTH SDS, and BA − CA, between the short, normal-weight, and the over-weight/obese subjects. The lack of significant differences in these parameters suggests that short obese children who were candidates for GHST had similar auxological characteristics as their normal-weight peers.

These findings are consistent with reports from the National Cooperative Growth Study Substudy IV showing: (1) no consistent differences in mean height SDS, BMI SDS, and GV SDS between patients with classic GHD and non-GHD short children; and (2) only small or no differences in the levels of IGF-I and IGFBP-3 between the patients selected for GH treatment and those who were not (27). Our findings are also similar to reports of an inverse relationship between BMI SDS and peak GH levels (5, 6), as well as studies showing that adiposity explains about 20% of the variability in peak GH levels (5, 7). The lack of auxological distinction between the GHD and non-GHD subjects might be due to the fact that the GHST is an imprecise test as peak GH response is influenced by several factors including nutrition (28), pubertal status (29), genes (30), and the occurrence of endogenous GH peak just before GHST (31).

The above finding of auxological similarity between short, normal-weight, and over-weight/obese children undergoing GHST differs from reports showing that obese children are generally taller than their non-obese peers (1–3). These studies report that obese children present with higher GV SDS and accelerated BA compared to non-obese children during the prepubertal years (4). Subsequently, this prepubertal advantage in GV decreases during puberty, with obese children showing a reduced growth spurt compared to lean subjects (32). This results in similar near adult heights in both the obese and non-obese children (33). There were non-significant differences in BA, or GV SDS between the over-weight/obese children and their normal-weight peers. There was equally no significant relationship between GV SDS and BA. The lack of auxological distinction between the over-weight/obese children and their normal-weight peers could also have arisen from differences in the timing of the onset of obesity as some studies have suggested that while early onset of obesity in the first 2 years of life is associated with tall stature, a later onset of obesity is not associated with tall stature (34).

The mechanism for the increased GV in childhood obesity is unknown (35). Some reports suggest that nutrition (36), serum concentrations of growth hormone binding protein (GHBP) (37), leptin (38), and insulin (39) could be involved in this phenomenon. Childhood obesity is characterized by normal or accelerated growth in spite of abnormalities of the GH/IGF-I axis, which are marked by reduced GH secretion with normal IGF-I levels compared to normal-weight peers (32). Additionally, in concert with leptin, increased insulin action on the IGF-I receptor has been suggested as a possible mechanism for the so called growth without GH syndrome (39).

There are a number of limitations to be considered in the interpretation of these results. The first limitation is the cross-sectional design of the study, which makes it impossible to establish causality. None of our subjects received sex-steroid priming before GHST. Even though this procedure is recommended as a tool to prevent false-positive results during GHST, it is not widely employed in clinical practice. The use of arginine and clonidine as the primary GH secretagogues in our study may have increased the proportion of false-positive cases as these agents are generally regarded as weak secretagogues. However, clonidine and arginine are the most widely used secretagogues in clinical practice because the powerful secretagogues are either discontinued (e.g., GHRH, l-DOPA) or are too dangerous to be employed in routine pediatric clinical practice (e.g., insulin).

Another limitation is the lack of the use of an adjusted cut-off value for the second phase of our sequential GH stimulation testing protocol. This could, theoretically, have resulted in a high prevalence of false-positive results as the second test is not independent of the first test and thus the somatotrophs may be refractory to the effects of the second secretagogue.

Another limitation is the lack of further testing with pyridostigmine in our cohort of obese children with GHD. Pyridostigmine is a cholinergic agonist that acts similar to arginine to suppress somatostatin release by the hypothalamus. This enhances GH response to GHRH both in normal subjects, and in many instances of impaired GH secretion, including obesity (40). It is usually administered in conjunction with GHRH during GH stimulation testing. Some investigators recommend that the diagnosis of GHD can only be made in an obese child in the presence of low height velocity, low IGF-I concentrations, and a subnormal peak GH response to standard stimulation that does not reverse with pyridostigmine administration. This additional testing with pyridostigmine is deemed necessary to prevent false-positive results because obese individuals are believed to have impaired hypothalamic secretion of GHRH, but possess a normal GH pituitary reserve (40). However, GHRH is no longer available in the United States, and the use of pyridostigmine is limited by its adverse effect profile as serious cholinergic side effects ranging from transient abdominal pain, muscle fasciculation, hypotension, and bradycardia have been reported in up to 23% of patients who received pyridostigmine during GHST (41).

We did not conduct a comprehensive biochemical evaluation of the hypothalamic–pituitary–adrenal axis in our short obese cohort. Such an evaluation is necessary because hypercortisolism arising from either iatrogenic or endogenous causes, e.g., Cushing disease, could easily lead to obesity and short stature, as a consequence of the impairment of GH secretion by cortisol. Thus, elevated levels of corticosteroids may inhibit or blunt GH response to GH secretagogues resulting in false-positive results during GHST. Our patients neither had a history of exposure to corticosteroids, nor showed any clinical signs suggestive of Cushing’s syndrome. Adrenal insufficiency, on the other hand, is not associated with obesity, but could result in growth impairment. Insulin induced hypoglycemia is employed in the simultaneous assessment of the functional reserves of both the hypothalamic–pituitary–adrenal axis and the GH/IGF-I axis (42). This test, however, is not routinely performed in clinical practice because of the associated risks of hypoglycemia and death.

This study also has several strengths. We had a relatively large sample size of prepubertal children to enable us to detect subtle differences between the groups of interest. All subjects received similar GH secretagogues, thus eliminating the wide variability in peak GH response that is associated with the use of secretagogues of differing potencies. The availability of data on GV SDS, MPTH SDS, child height SDS minus MPTH SDS, and BA − CA enabled us to test the hypothesis that obese children were overdiagnosed with GHD in a novel and rigorous manner. All our results were adjusted for possible confounders.

## Conclusion

Though this study found evidence for reduced GH secretion during GHST in short, prepubertal, over-weight/obese children compared to their short normal-weight peers, there were no evidence for higher GV SDS, child height minus MPTH SDS, accelerated BA, or elevated levels of IGF-I, or IGFBP-3 in the over-weight/obese children compared to their normal-weight peers. The absence of adiposity-related auxological differences in short obese children suggests that pre-GHST auxological characteristics may not be sensitive in excluding false-positive cases of GHD.

## Conflict of Interest Statement

The authors declare that the research was conducted in the absence of any commercial or financial relationships that could be construed as a potential conflict of interest.
